# Longitudinal Associations of Newly Diagnosed Prediabetes and Diabetes with Cognitive Function among Chinese Adults Aged 45 Years and Older

**DOI:** 10.1155/2022/9458646

**Published:** 2022-07-28

**Authors:** Xiaojie Wang, Xiuwen Li, Wanxin Wang, Guangduoji Shi, Ruipeng Wu, Lan Guo, Ciyong Lu

**Affiliations:** ^1^Department of Medical Statistics and Epidemiology, School of Public Health, Sun Yat-sen University, Guangzhou 510080, China; ^2^Department of Neurology, Shenzhen Qianhai Shekou Free Trade Zone Hospital (Shenzhen Shekou People's Hospital), Shenzhen 518067, China

## Abstract

With population aging, diabetes mellitus and cognitive function decline are common health problems among older adults worldwide. This longitudinal study is aimed at estimating the longitudinal associations of newly diagnosed prediabetes and diabetes status with cognitive function among Chinese adults aged 45 years and older and evaluating the clinical risk factors associated with cognitive function. Data were obtained from the China Health and Retirement Longitudinal Study (CHARLS). A total of 8716 participants meeting the inclusion criteria were enrolled between 2011 and 2012 at baseline, and 6125 participants completed the follow-up survey in 2018. Cognitive function, newly diagnosed diabetic status, depression, body mass index, and clinical and biochemical measurements were collected. At baseline, the mean age of the participants was 58.93 (SD: 9.76) years, 3987 (45.7%) were males, 1802 (20.7%) participants were newly diagnosed with prediabetes, and 935 (10.7%) were diabetes patients. After adjusting for control variables, diabetes was a significant risk factor for subsequent cognitive decline (unstandardized *β*estimate = −0.50, 95%CI = −0.98 ~ −0.02). Subgroup analyses found that the association of diabetes with cognitive decline was significant in females. Stratification analyses found that among prediabetes patients, triglyceride concentrations were negatively associated with cognitive function; among diabetes patients, high-sensitivity C-reactive protein was significantly associated with cognitive decline. The newly diagnosed diabetes status at baseline was associated with subsequent cognitive decline among middle-aged and elderly Chinese, especially in females. The management of triglycerides through lifestyle modification for prediabetes and specific adjunctive anti-inflammatory therapy for diabetes might benefit cognitive performance.

## 1. Introduction

With population aging, diabetes mellitus is one of the most common problems among older adults worldwide [1]. China is one of the top three countries globally with the diabetes epidemic, and it continues to increase with demographic and social transitions due to rapid aging, urbanization, and lifestyle change [2]. Moreover, prediabetes, which is an intermediate metabolic state of hyperglycemia higher than normal but lower than the clinical diabetes threshold, may progress to diabetes as high as 74% [3]. According to recent data from a nationally representative cross-sectional survey in China, diabetes prevalence in Chinese adults is 10.9%, and prediabetes prevalence is nearly 35.7% [4]. Besides, a recent national study also reported that the prevalence of total diabetes, self-reported diabetes, newly diagnosed diabetes, and prediabetes diagnosed by the American Diabetes Association (ADA) criteria was 12.8%, 6.0%, 6.8%, and 35.2%, respectively, among adults living in China [5]. As a glucose metabolism disorder, prediabetes and diabetes have already been known to be associated with a variety of clinical sequelae, including vascular and nonvascular diabetic-related complications, resulting in an elevated risk of death and health costs [6, 7]. Moreover, diabetes has been reported to be a risk factor for cognitive function decline, cognitive function impairment, or even dementia (i.e., the most serious stages in the development of cognitive dysfunction) [8, 9].

Cognitive function decline or impairment significantly elevates the risk of poor quality of life in older adults and most possibly occurs with aging [10]. However, although cognitive decline among older adults is common, it is often overlooked for early identification or even progress to dementia, which has burdened individual families and society [11]. In particular, in Chinese culture, older adults have culturally various perspectives on cognitive function decline or impairment than their counterparts in Western culture, and cognitive function impairment is culturally stigmatized or socially discriminated [12]. Previous evidence suggests a significant correlation between physical and cognitive impairment in diabetic elderly patients [13], and odor identification, knee extension strength, and balance capability were potential markers for cognitive decline in middle-aged persons with diabetes (Midorikawa et al., 2021). Although some studies reported that prediabetes or diabetes in midlife was associated with a more significant cognitive decline [14] and may decrease the probability of mild cognitive impairment reversion [15], the causative mechanisms of diabetes on cognitive impairment are still unclear [16]. A recent umbrella review suggested that prediabetes was associated with a higher risk of all-cause dementia (e.g., Alzheimer's or vascular). However, no significant associations between prediabetes and mild cognitive impairment were observed [17]. Then, the associations between prediabetes and cognitive function are required to be further elucidated. Besides, little research explores the effects of the onset of prediabetes and diabetes on subsequent cognitive function among Chinese adults, especially considering the differences in the associations between diabetes and cognitive function decline according to the biological sex or age group.

Previous evidence showed that there might be shared inflammatory pathways in relation to insulin resistance and cognitive impairment [18]; a prior study also reported that elevated triglycerides (TG) were associated with smaller brain volume (i.e., correlated with general cognitive ability), even in patients without diabetes [19], suggesting that there might be different mechanisms in the association of prediabetes and diabetes with cognitive function decline, respectively. Therefore, this study, using data from the China Health and Retirement Longitudinal Study (CHARLS), is aimed at (1) estimating the longitudinal associations of newly diagnosed prediabetes and diabetes status with cognitive function among Chinese adults aged 45 years and older during an 8-year period, with a particular focus on different biological sex and age group and (2) estimating the clinical risk factors associated with cognitive function among patients with prediabetes and diabetes.

## 2. Methods

### 2.1. Study Design and Participants

**Figure 1:**
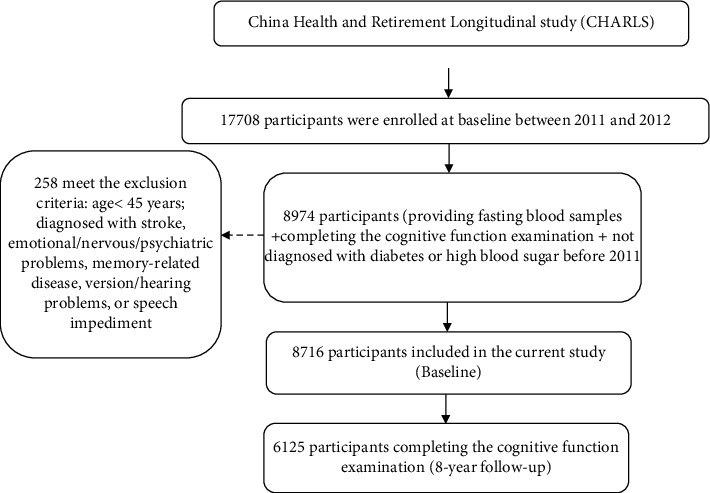
Flowchart of the study using data from the China Health and Retirement Longitudinal Study.

Data were obtained from the CHARLS, which used a multistage probability sampling method to recruit Chinese residents aged 45 and older from 28 of the overall provinces in China. The details of the study sampling method have been reported elsewhere [20]. The current study was the secondary analysis of the baseline data (Wave 1) and Wave 4 follow-up data in 2018 (Wang *et al.*). As shown in Figure 1, among the surveyed participants at baseline, 8974 participants who provided fasting blood samples, had not been diagnosed with diabetes or high blood sugar before, and completed the cognitive function examination, were selected. After excluding 258 individuals aged <45 years and those who have been diagnosed with stroke, emotional/nervous/psychiatric problems, memory-related disease, vision/hearing problems, or speech impediment, 8716 participants were included at baseline in the study, and 6125 participants who completed the cognitive function examination were eligible for the follow-up survey over an 8-year period (in 2018; retention rate: 70.3%) [21]. Each participant provided written informed consent before participating in this study. Ethics approval for the data collection of the CHARLS study was obtained from the Biomedical Ethics Review Committee of Peking University (IRB00001052-11015). Ethics approval for the use of CHARLS data was obtained from the University of Newcastle Human Research Ethics Committee. A comparison between the final sample and the loss of follow-up samples in the baseline characteristics is presented in Supplementary Table 1. In this study, the loss to follow-up samples represented a higher proportion of females, widowed, with lower education level, and self-reported poor health; the loss to follow-up sample represented an older age [22].

### 2.2. Data Collection

#### 2.2.1. Questionnaire

A standard questionnaire administered by trained staff was used to collect information. Sociodemographic characteristics, including sex, age, marital status (1 = married, 2 = separated or divorced, 3 = widowed, and 4 = never married), education level (1 = primary school or below, 2 = middle school, and 3 = high school or above), ever smoking (1 = yes, 2 = no), and ever drinking (1 = yes, 2 = no), were collected. Self-comment about health was assessed by asking how about your health status comparing your peers or friends (responses were classified into 1 = good, 2 = fair, and 3 = poor)? Hypertension was assessed by asking, “have you been told that you have hypertension by a doctor before 2011 (responses included 1 = yes and 2 = no)”? Dyslipidemia was measured by asking, “have you been told that you have dyslipidemia by a doctor before 2011 (responses included 1 = yes and 2 = no)”?

#### 2.2.2. Depressive Symptoms

Depressive symptoms were measured by the 10-item Center for Epidemiology Scale for Depression (CESD-10) in Chinese [23], which has been validated and extensively used among Chinese adults [24]. The sum of scores ranges from 0 to 30, with higher scores indicating a higher level of depressive symptom severity.

#### 2.2.3. Cognitive Function

In this study, the primary outcomes were general cognition functioning. The Telephone Interview of Cognitive Status (TICS-10; orientation and attention), word recall (episodic memory), and figure drawing (visual-spatial abilities) were used to assess cognitive functioning, with an overall cognition score incorporating these assessments.

The overall cognitive functioning score ranged from 0 to 21, and a higher score indicated better cognitive performance ([25, 26]. The TICS-10 used in this study consists of ten mental status questions: the orientation of the date (months, day, and year), the orientation of days of the week, the orientation of seasons of the year, and serial subtraction of 7 from 100 (up to five times) [27]. The TICS-10 score was the total number of correct answers and ranged from 0 to 10 [2]. Regarding word recall, participants were first given about two minutes to immediately recall as many words as they could in any order after the interviewers read a list of 10 Chinese words (immediate recall). About four to ten minutes later, participants were asked to recall as many original words as possible (delayed recall). The word recall score consisted of the average number of immediate and delayed word recalls and ranged from 0 to 10 [25]. Regarding figure drawing, participants were asked to draw a similar figure of two pentagons overlapped with each other. Participants who completed this task received a score of 1 and 0 if they failed to do so [28].

#### 2.2.4. Clinical and Biochemical Measurements

A 4 mL sample of whole blood was collected to obtain plasma and buffy coat, and another 2 mL sample of whole blood was collected for HbA_1C_ analysis. All blood samples were stored in a local laboratory at 4°C and were transported at -80°C to the China Center of Disease Control (CDC) in Beijing within two weeks. High-sensitivity C-reactive protein (Hs-CRP), HbA_1C_, a lipid panel (total, HDL, LDL cholesterol, and TG), glucose, blood urea nitrogen (BUN), creatinine, and cystatin C from frozen plasma or whole blood samples were measured. Hs-CRP level was assessed by immunoturbidimetric assay. HbA_1c_ levels were determined using Boronate affinity high-performance liquid chromatography (HPLC). Total cholesterol, HDL cholesterol (HDL-c), LDL cholesterol (LDL-c), TG, and FBG concentrations were measured using enzymatic colorimetric tests. Blood urea nitrogen (BUN) level was assessed by the enzymatic UV method with urease. Creatinine concentration was measured using the rate-blanked and compensated Jaffe creatinine method. Cystatin C level was evaluated by using a particle-enhanced turbidimetric assay [29].

#### 2.2.5. Diabetic Status

Participants were first assessed by asking the question “have you been told that you have diabetes by a doctor before 2011?” Considering cognitive functioning might be influenced by a long-term disease or treatment, we focused on newly diagnosed diabetic status in this study. Those diagnosed with diabetes before or without providing fasting blood samples were excluded. Diabetic status was assessed according to the 2010 ADA guidelines. Prediabetes was defined as a fasting blood glucose (FBG) level of 100-125 mg/dL or glycated hemoglobin (HbA_1c_) level of 5.7-6.4%; diabetes was defined as an FBG level ≥ 126 mg/dL or an HbA_1c_ level ≥ 6.5% [30].

### 2.3. Statistical Analysis

Baseline sample characteristics were described separately in both total adults and based on diabetic status. Categorical data were reported as frequencies (%); normally distributed continuous variables were presented as mean (±SD), and skewed data were presented as medians (interquartile range). The Rao-Scott chi-square test for categorical variables and the one-way ANOVA test for continuous variables were used to assess the differences between different diabetic statuses. Univariable generalized mixed-effects linear regression models were performed to estimate the baseline factors associated with subsequent cognitive function scores. Multivariable generalized mixed-effects linear regression models were conducted to estimate the longitudinal associations of prediabetes and diabetes with cognitive function decline. Factors associated with diabetic status or cognitive function in the univariable analyses or widely reported were considered control variables. In addition, subgroup analyses were performed according to biological sex and age to estimate whether the associations of prediabetes and diabetes with cognitive function scores were robust in different sexes or age groups. Furthermore, multivariable generalized mixed-effects linear regression models stratified by diabetic status were performed to investigate the clinical factors associated with cognitive function among patients with prediabetes or diabetes. All statistical analyses were conducted using Stata 16.0 SE (StataCorp, Houston, Texas, USA). Statistical significance was evaluated at the <0.05 level (two-tailed).

## 3. Results

### 3.1. Baseline Characteristics of Participants with Different Diabetic Statuses

Table 1 summarizes the baseline characteristics of the 8716 participants. The mean age of the participants was 58.93 (SD: 9.76) years, and 3987 (45.7%) were males. Notably, 1802 (20.7%) participants were newly diagnosed with prediabetes at baseline, and 935 (10.7%) were newly diagnosed diabetes patients. The variances between the different diabetic groups were statistically significant in the distribution of age, marital status, ever smoking, ever drinking, self-comment about health, hypertension, dyslipidemia, cognitive function, BMI, FBG, HbA_1c_, BUN, creatinine, total cholesterol, TG, HDL-c, LDL-c, Hs-CRP, and hemoglobin (*P* < 0.05).

### 3.2. Factors Associated with Cognitive Function at Follow-Up

Without adjusting for other variables, diabetes status was a risk factor for subsequent cognitive decline (unstandardized *β* estimate = −0.60, 95%CI = −1.05 ~ −0.16). In contrast, the association between baseline prediabetes and subsequent cognitive function was not significant (Supplementary Table 2). Other variables associated with subsequent cognitive function are also presented in Supplementary Table 2, including questionnaire information (i.e., sex, age, marital status, education level, ever smoking, ever drinking, self-comment about health, dyslipidemia, depressive symptoms, and cognitive functioning at baseline) and clinical and biochemical measurements (i.e., BMI, BUN, creatinine, TG, HDL-c, Hs-CRP, hemoglobin, and cystatin C).

### 3.3. Eight-Year Association between Baseline Diabetic Status and Subsequent Cognitive Function

As shown in Table 2, after adjusting for age, gender, marital status, education level, ever smoking, ever drinking, self-comment about health, hypertension, dyslipidemia, BMI, and depressive symptoms at baseline, newly diagnosed diabetes patients were at a higher risk of cognitive decline at follow-up (unstandardized *β* estimate = −0.57, 95%CI = −0.96 ~ −0.18, model 1). However, after adjusting for variables in model 1 plus cognitive function at baseline, the significant association vanished (*P* > 0.05, model 2). After further adjusting for the variables in model 2 plus BUN, creatinine, TG, HDL-c, LDL-c, Hs-CRP, hemoglobin, cystatin C, and HbA_1c_, diabetes status was a significant risk factor for subsequent cognitive decline (unstandardized *β* estimate = −0.50, 95%CI = −0.98 ~ −0.02, model 3).

### 3.4. Subgroup Analyses

As shown in Table 3, participants were divided into different groups based on biological sex and age status. The subgroup analysis found differences between males and females in the longitudinal associations of prediabetes and diabetes with cognitive decline. Only for females, the adjusted association of newly diabetes (unstandardized *β* estimate = −0.75, 95%CI = −1.43 ~ −0.07, model 3) with cognitive decline was statistically significant.  Table 4 shows the subgroup analyses according to age status. However, no difference in the associations of diabetic status with cognitive function was observed between the individuals 45 years ≤ baseline age < 60 years and those baseline age ≥ 60 years, and no significant association was found in the two groups.

### 3.5. Clinical Characteristics Associated with Cognitive Function

Table 5 shows that after adjusting for age, gender, marital status, education level, ever smoking, ever drinking, self-comment about health, hypertension, dyslipidemia, BMI, depressive symptoms, and cognition function at baseline, the models showed that TG concentrations were negatively associated with cognitive function among prediabetes patients (unstandardized *β* estimate = −0.004, 95%CI = −0.007 ~ −0.001), and Hs-CRP was significantly associated with cognitive decline among diabetes patients (unstandardized *β* estimate = −0.065, 95%CI = −0.122 ~ −0.009).

### 3.6. Sensitivity Analyses

We also used the 1999 World Health Organization (WHO) diagnostic criteria for diabetic status to assess the robustness of our findings on the association between baseline diabetic status and cognitive function at an 8-year follow-up. In the WHO criteria, diabetes status is defined as an FBG level ≥ 126 mg/dL, and impaired fasting glucose (IFG) is defined as an FBG level of 110-125 mg/dL. As shown in Supplementary Table 3, the sensitivity analyses were consistent with the primary analyses, suggesting that baseline diabetes status was significantly associated with subsequent cognitive decline (unstandardized *β* estimate = −0.57, 95%CI = −1.05 ~ −0.10, model 4).

## 4. Discussion

This longitudinal study observed that at baseline, the prevalence of newly diagnosed prediabetes and diabetes among Chinese adults aged 45 years and older was 20.7% and 10.7%, respectively. This finding was consistent with previous studies [4, 31], suggesting that prediabetes or diabetes has been a significant public health problem among Chinese adults. First, the univariable analyses found that except for diabetic status, males, age, widowed or never married, ever smoking, poor self-comment about health, hypertension, dyslipidemia, and depressive symptoms scores at baseline were negatively associated with subsequent cognitive function. These findings might help identify a profile of adults at higher risk of cognitive decline and provide potential confounders that may affect the association between diabetes status and cognitive function. Moreover, without adjusting for other variables, univariable analyses also observed that patients with newly diagnosed diabetes were at a higher risk of cognitive decline at 8-year follow-up; nevertheless, baseline prediabetes status was not significantly associated with subsequent cognitive function decline. By extensively adjusting for age, gender, marital status, education level, ever smoking, ever drinking, self-comment about health, hypertension, dyslipidemia, BMI, and depressive symptoms at baseline, this longitudinal study found that the newly diagnosed diabetes status at baseline still predicted subsequent cognitive decline. Similarly, previous cross-sectional and longitudinal studies have suggested that diabetes status was associated with a higher risk of cognitive decline among adults and older adults [9, 32, 33], and prediabetes was not related to poorer cognitive performance among general older adults or patients after stroke [34, 35]. Several possible biological explanations have been proposed, including the indirect effects of diabetes on cognition through subclinical or clinical vascular disease, in that diabetes can cause damage to cerebral microvascular and macrovascular, contributing to cognitive decline [36, 37]. Previous evidence also suggests microvascular dysfunction is a widespread phenomenon in people with diabetes, and cerebral microvascular dysfunction is also apparent in adults with prediabetes; hyperglycemia, obesity and insulin resistance, and hypertension are main drivers of diabetes-related cerebral microvascular dysfunction, and increasing amounts of data from observational studies have suggested that diabetes-related microvascular dysfunction is associated with a higher risk of cognitive dysfunction [38]. Another explanation might be related to the abnormal insulin modification in diabetes patients. In the central nervous system, insulin plays a critical regulatory role. At the same time, hyperglycemia can lead to the accumulation of advanced glycation and products (i.e., the primary contributor to insulin resistance in diabetic cells), and brain insulin resistance is a key factor in the pathogenesis of Alzheimer's disease by interacting with key proteins affected in neurodegenerative conditions (e.g., amyloid-beta precursor protein) [39, 40]. Furthermore, diabetes is a risk factor for frailty, a multidimensional condition for reserve loss and susceptibility to stressors with a high risk of death, hospitalization, and functional and cognitive impairment [13, 41, 42]. Moreover, previous evidence has reported that cognitive declines occur with normal aging for structural and functional changes in the brain, including loss of synapses, alterations in neuronal structure without neuronal death, and dysfunction of neuronal networks. Age-related diseases may also accelerate the rate of neuronal loss, neuronal dysfunction, and cognitive declines [43]. Although this study also observed a negative association between age and subsequent cognitive function and the observed associations between baseline diabetic status and subsequent cognitive function were adjusted for age, we cannot rule out the impact of age on the association between diabetic status and cognitive function.

Further adjusting for cognitive function at baseline, the significant association disappeared, indicating that baseline cognitive function was the most vital factor associated with subsequent cognitive function. Nevertheless, after adding clinical and biochemical factors (e.g., BUN, creatinine, TG, HDL-c, LDL-c, Hs-CRP, hemoglobin, cystatin C, and HbA_1c_) as the control variables, the multivariable models observed that newly diagnosed diabetes status at baseline was associated with a 0.50-fold increase in the risk of cognitive function decline, suggesting that there might be various clinical and biochemical factors related to cognitive function among individuals with different diabetic status.

Furthermore, subgroup analyses based on biological sex and age status showed that after adjusting for control variables (including questionnaire information and clinical and biochemical measurements), newly diagnosed diabetes was a risk factor for subsequent cognitive decline in females. Similarly, Chatterjee et al. reported that for vascular dementia (not for nonvascular dementia), the additional risk of diabetes is more significant in women [44]. Our further stratification analyses according to diabetic status found that higher triglyceride concentration was a risk factor for cognitive function among prediabetes patients. Similarly, Power et al. reported that elevated serum TGs were associated with a greater 20-year decline in cognitive function by using a cohort study of persons recruited at ages 45 to 65 years from U.S. communities [45]; He et al. found a significant association between high plasma TG levels and mild cognitive impairment among participants aged >65 years [46]. A possible explanation was that higher levels of TG might increase global cerebral amyloid beta deposition affecting cognitive function transition, and another explanation may be that higher TG level was a risk factor for cerebrovascular disease, which may cause cognitive decline through hypoperfusion [47]. Furthermore, the stratification analyses also revealed that among diabetes patients, Hs-CRP level was negatively associated with subsequent cognitive function. These findings were consistent with previous longitudinal studies, which suggest that Hs-CRP levels were positively related to future cognitive impairment and decline in elderly individuals with cardiovascular disease [48] and euthymic patients with bipolar disorder [49]. These findings might be related to that Hs-CRP is a vital biomarker for systemic inflammation, and elevation of peripheral inflammation may activate the central nervous system, including brain microglia, serotonin transporter expression, oxidative stress, and decreased neuroplasticity, all potentially contributing to structural and functional brain changes, which all with accumulation can cause cognitive performance-related disease [50]. Besides, evidence also suggests that there might be shared inflammatory pathways concerning insulin resistance and cognitive impairment [18]. To sum up, this study suggested that the management of TG through lifestyle modification (e.g., increasing physical activity and improving dietary intake according to the corresponding guideline) or specific therapy could bring benefits to cognitive performance among prediabetes patients [51]. Another potential clinical implication is that adjunctive anti-inflammatory treatments may improve cognitive function among diabetes patients.

The strengths of the current study included adopting a large-scale, 8-year longitudinal study design, using the new diagnosis of prediabetes and diabetes as exposure, and the use of questionnaires and clinical and biochemical measurements to collect information. Several potential limitations should also be notable. First, although data about cognitive function was measured by self-report, which may lead to biased reporting, self-reports remain a common and accepted method for assessing cognitive performance. Second, the study sample only included adults aged 45 years and older, and then, the generalization of the findings may not be applicable to all Chinese adults. Third, although this study adopted an eight-year longitudinal study design, the associations should still be interpreted cautiously because they were generated from an interval that might not be long enough to uncover apparent cognitive decline. Fourth, although a sensitivity analysis performed using another WHO diagnostic criteria for diabetic status yielded the same result as the original analyses, the associations of prediabetes and diabetes status with cognitive function should also be exclusively analyzed in confirmed cases in future studies. Fifth, the prevalence of undiagnosed diabetes in China is considerable, especially in rural China. Although this study only included newly diagnosed diabetes, undiagnosed and uncontrolled long-term diabetes may exist in the newly diagnosed diabetic group, leading to an overestimating of the association between diabetic status and cognitive function. Sixth, previous evidence reported that older people with diabetes receiving antidiabetic treatment (i.e., metformin use) have slower cognitive decline and lower dementia risk [52]. However, detailed information about antidiabetic treatment after newly diagnosed with diabetes at baseline is unavailable in this study.

## 5. Conclusion

In conclusion, this longitudinal study observed significant associations between baseline newly diagnosed diabetes status and cognitive decline at 8-year follow-up among Chinese adults aged 45 years and older, especially in females. Although this study did not find statistically significant associations between baseline prediabetes status and subsequent cognitive function, patients with prediabetes have a higher risk of developing diabetes. Moreover, the stratification analysis according to diabetic status reported that among the prediabetes group, TG level was negatively associated with cognitive function; among diabetes patients, higher Hs-CRP levels predicted an elevated risk of cognitive decline.

## Figures and Tables

**Table 1:** Baseline characteristics of participants with different diabetic statuses.

Variables	Baseline survey (*n* = 8716)	Baseline diabetic status^a^			*P* value^c^
		Prediabetes (*n* = 1802)	Diabetes (*n* = 935)	Others^b^ (*n* = 5979)	
Gender					
Male	3987 (45.7)	849 (21.3)	444 (11.1)	2694 (67.6)	0.165
Female	4717 (54.1)	952 (20.2)	489 (10.4)	3276 (69.5)	
Missing data	12 (0.1)				
Age^∗^ (year)	58.93 (9.76)	59.70 (9.53)	60.39 (10.00)	58.48 (9.76)	<0.001
Marital status					
Married	7690 (88.2)	1559 (20.3)	815 (10.6)	5316 (69.1)	0.029
Separated or divorced	95 (1.1)	19 (20.0)	7 (7.4)	69 (72.6)	
Widowed	869 (10.0)	208 (23.9)	107 (12.3)	554 (63.8)	
Never married	52 (0.6)	15 (28.8)	4 (7.7)	33 (63.5)	
Missing data	10 (0.1)				
Education level					
Primary school or below	5953 (68.3)	1236 (20.8)	641 (10.8)	4076 (68.5)	0.945
Middle school	1797 (20.6)	375 (20.9)	192 (10.7)	1230 (68.4)	
High school or above	948 (10.9)	188 (19.8)	98 (10.3)	662 (69.8)	
Missing data	18 (0.2)				
Ever smoking					
Yes	3352 (38.5)	728 (21.7)	381 (11.4)	2243 (66.9)	0.028
No	5362 (61.5)	1074 (20.0)	554 (10.3)	3734 (69.6)	
Missing data	2 (0)				
Ever drinking					
Yes	5357 (61.5)	1073 (20.0)	550 (10.3)	3734 (69.7)	0.018
No	3359 (38.5)	729 (21.7)	385 (11.5)	2245 (66.8)	
Self-comment about health					
Good	1664 (19.1)	298 (17.9)	208 (12.5)	1158 (69.6)	0.004
Fair	5484 (62.9)	1168 (21.3)	552 (10.1)	3764 (68.6)	
Poor	1568 (18.0)	336 (21.4)	175 (11.2)	1057 (67.4)	
Hypertension					
Yes	2034 (23.3)	500 (24.6)	275 (13.5)	1259 (61.9)	<0.001
No	6651 (76.3)	1298 (19.5)	658 (9.9)	4695 (70.6)	
Missing data	31 (0.4)				
Dyslipidemia					
Yes	689 (7.9)	178 (25.8)	95 (13.8)	416 (60.4)	<0.001
No	7894 (90.6)	1591 (20.2)	831 (10.5)	5472 (69.3)	
Missing data	133 (1.5)				
Depressive symptoms^∗^ (CESD-10)	9.06 (5.37)	9.05 (5.42)	8.72 (5.46)	9.12 (5.33)	0.105
Cognitive functioning^∗^ (overall)	9.71 (5.07)	9.64 (5.08)	9.30 (5.17)	9.80 (5.04)	0.023
TICS^∗^	5.56 (3.83)	5.55 (3.82)	5.31 (3.93)	5.59 (3.82)	0.109
Word recall^∗^	3.09 (2.00)	3.03 (1.96)	2.83 (2.05)	3.15 (2.00)	<0.001
Complete figure drawing (%)	5267 (60.4)	1082 (20.5)	534 (10.1)	3651 (69.3)	0.192
BMI^∗^ (kg/m^2^)	23.43 (3.60)	23.95 (3.77)	24.20 (3.77)	23.15 (3.49)	<0.001
FBG^∗^ (mg/dL)	106.81 (29.11)	113.25 (7.92)	160.78 (60.33)	96.42 (8.50)	<0.001 (60.33)
HbA_1c_^∗^ (%)	5.21 (0.66)	5.34 (0.47)	6.03 (1.42)	5.04 (0.34)	<0.001
BUN^∗^ (mg/dL)	15.66 (4.55)	16.02 (4.90)	16.00 (4.79)	15.50 (4.39)	<0.001
Creatinine^∗^ (mg/dL)	0.78 (0.24)	0.80 (0.35)	0.79 (0.21)	0.77 (0.19)	<0.001
Total cholesterol^∗^ (mg/dL)	193.75 (38.86)	200.56 (40.38)	204.21 (46.96)	190.06 (36.35)	<0.001
TG^∗^ (mg/dL)	129.66 (103.6)	147.04 (100.97)	207.56 (217.60)	112.24 (61.86)	<0.001
HDL-c^∗^ (mg/dL)	51.54 (15.15)	50.52 (15.90)	47.01 (16.65)	52.56 (14.51)	<0.001
LDL-c^∗^ (mg/dL)	117.21 (34.74)	120.49 (37.10)	114.28 (41.13)	116.67 (32.81)	<0.001
Hs-CRP^#^ (mg/dL)	1.01 (1.57)	1.15 (1.81)	1.40 (2.31)	0.90 (1.37)	<0.001
Hemoglobin^∗^ (g/dL)	14.41 (2.25)	14.57 (2.11)	14.57 (2.36)	14.33 (2.27)	<0.001
Cystatin C^∗^ (mg/L)	1.02 (0.28)	1.02 (0.35)	1.01 (0.28)	1.02 (0.26)	0.855

Abbreviation: CESD-10: the 10-item Center for Epidemiology Scale for Depression; BMI: body mass index; BUN: blood urea nitrogen; TG: triglyceride; HDL-c: HDL cholesterol; LDL-c: LDL cholesterol; Hs-CRP: high-sensitivity C-reactive protein. ^∗^Data was presented in mean (SD). ^#^Data was described in median (interquartile range). ^a^Diabetic status was newly diagnosed prediabetes or diabetes assessed according to the 2010 American Diabetes Association (ADA) guidelines. ^b^Others: individuals without prediabetes or diabetes. ^c^The Rao-Scott chi-square test for categorical variables and one-way ANOVA test for continuous variables were used to assess the differences between the groups.

**Table 2:** Eight-year association between baseline diabetic status and subsequent cognitive function (multivariable analyses).

Baseline diabetic status	Cognitive function (follow-up, *n* = 6125)					
	Model 1		Model 2		Model 3	
	Unstandardized *β* estimate (95% CI)	*P* value	Unstandardized *β* estimate (95% CI)	*P* value	Unstandardized *β* estimate (95% CI)	*P* value
Others^a^	Ref.		Ref.		Ref.	
Prediabetes	-0.006 (-0.29~0.28)	0.965	0.009 (-0.26~0.27)	0.944	0.03 (-0.28~0.35)	0.839
Diabetes	-0.57 (-0.96~-0.18)	0.004	-0.31 (-0.67~0.04)	0.083	-0.50 (-0.98~-0.02)	0.041

Abbreviation: 95% CI: 95% confidence interval; Ref: reference. ^a^Others, individuals without prediabetes or diabetes. Model 1: adjusting for age, gender, marital status, education level, ever smoking, ever drinking, self-comment about health, hypertension, dyslipidemia, BMI, and depressive symptoms at baseline. Model 2: adjusting for the variables in model 1 plus cognitive function at baseline. Model 3: adjusting for the variables in model 2 plus clinical variables including blood urea nitrogen, creatinine, triglycerides, HDL cholesterol, LDL cholesterol, high-sensitivity C-reactive protein, hemoglobin, cystatin C, and HbA_1c_.

**Table 3:** Eight-year association between baseline diabetic status and subsequent cognitive function according to biological sex.

Baseline diabetic status	Cognitive function (follow-up), unstandardized *β* estimate (95% CI)^#^					
	Female			Male		
	Model 1	Model 2	Model 3	Model 1	Model 2	Model 3
Others ^a^	Ref.	Ref.	Ref.	Ref.	Ref.	Ref.
Prediabetes	-0.26 (-0.66~0.14)	-0.17 (-0.53~0.19)	-0.30 (-0.74~0.13)	0.36 (-0.04~0.76)	0.31 (-0.07~0.68)	-0.32 (-0.99~0.36)
Diabetes	-0.72 (-1.26~-0.17)^∗^	-0.55 (-1.04~-0.06)^∗^	-0.75 (-1.43~-0.07)^∗^	-0.28 (-0.82~0.26)	0.003 (-0.07~0.68)	0.36 (-0.10~0.82)

Abbreviation: 95% CI: 95% confidence interval; Ref: reference. ^a^Others, individuals without prediabetes or diabetes. Model 1: adjusting for age, marital status, education level, ever smoking, ever drinking, self-comment about health, hypertension, dyslipidemia, BMI, and depressive symptoms at baseline. Model 2: adjusting for the variables in model 1 plus cognitive function at baseline. Model 3: adjusting for the variables in model 2 plus clinical variables including blood urea nitrogen, creatinine, triglycerides, HDL cholesterol, LDL cholesterol, high-sensitivity C-reactive protein, hemoglobin, cystatin C, and HbA_1C_. ^∗^*P* < 0.05.

**Table 4:** Eight-year association between baseline diabetic status and subsequent cognitive function according to age.

Baseline diabetic status	Cognitive function (follow-up), unstandardized *β* estimate (95% CI)					
	45 years ≤ baseline age < 60 years			Baseline age ≥ 60 years		
	Model 1	Model 2	Model 3	Model 1	Model 2	Model 3
Others^a^	Ref.	Ref.	Ref.	Ref.	Ref.	Ref.
Prediabetes	-0.08 (-0.43~0.26)	-0.22 (-0.65~0.22)	-0.56 (-1.16~0.05)	0.04 (-0.49~0.56)	0.17 (-0.29~0.62)	-0.52 (-1.33~0.30)
Diabetes	-0.39 (-0.86~0.07)	-0.07 (-0.39~0.25)	-0.18 (-0.59~0.23)	-1.09 (-1.82~-0.36)^∗^	-0.53 (-1.16~0.10)	0.26 (-0.26~0.78)

Abbreviation: 95% CI: 95% confidence interval; Ref: reference. ^a^Others, individuals without prediabetes or diabetes. Model 1: adjusting for age, marital status, education level, ever smoking, ever drinking, self-comment about health, hypertension, dyslipidemia, BMI, and depressive symptoms at baseline. Model 2: adjusting for the variables in model 1 plus cognitive function at baseline. Model 3: adjusting for the variables in model 2 plus clinical variables including blood urea nitrogen, creatinine, triglycerides, HDL cholesterol, LDL cholesterol, high-sensitivity C-reactive protein, hemoglobin, cystatin C, and HbA_1C_. ^∗^*P* < 0.05.

**Table 5:** Clinical characteristics associated with cognitive function among patients with different diabetic statuses.

Baseline characteristics	Cognitive function (follow-up, *n* = 6125)					
	Prediabetes		Diabetes		Others^a^	
	Unstandardized *β* estimate (95% CI)^#^	*P* value	Unstandardized *β* estimate (95% CI)^#^	*P* value	Unstandardized *β* estimate (95% CI)^#^	*P* value
BUN (mg/dL)^∗^	0.040 (-0.023~0.102)	0.210	-0.008 (-0.098~0.083)	0.863	0.014 (-0.023~0.050)	0.467
Creatinine (mg/dL)^∗^	-0.419 (-2.348~1.510)	0.670	-0.788 (-3.330~1.852)	0.591	0.690 (-0.398~1.777)	0.214
TG (mg/dL)^∗^	-0.004 (-0.007~-0.001)	0.037	0.001 (-0.004~0.002)	0.721	0.001 (-0.002~0.004)	0.397
HDL-c (mg/dL)∗	-0.006 (-0.026~0.015)	0.569	0.001 (-0.027~0.028)	0.969	-0.002 (-0.014~0.010)	0.743
LDL-c (mg/dL)^∗^	0.000 (-0.008~0.007)	0.921	0.002 (-0.009~0.013)	0.742	-0.001 (-0.005~0.004)	0.811
Hs-CRP (mg/dL)^∗^	-0.028 (-0.071~0.016)	0.210	-0.066 (-0.121~-0.012)	0.017	0.008 (-0.018~0.033)	0.556
Hemoglobin (g/dL)^∗^	-0.049 (-0.189~0.092)	0.497	0.026 (-0.169~0.221)	0.795	0.033 (-0.037~0.103)	0.353
Cystatin C (mg/L)^∗^	0.013 (-1.451~1.476)	0.987	0.892 (-1.379~3.163)	0.441	-0.757 (-1.513~-0.001)	0.050
HbA_1c_ (%)	0.629 (0.046~1.212)	0.034	-0.046 (-0.363~0.272)	0.795	0.021 (-0.417~0.458)	0.926

Abbreviation: BUN: blood urea nitrogen; TG: triglyceride; HDL-c: HDL cholesterol; LDL-c: LDL cholesterol; Hs-CRP: high-sensitivity C-reactive protein; 95% CI: 95% confidence interval. ^a^Others, individuals without prediabetes or diabetes. ^∗^Continuous variable with 1-unit increase. ^#^Models were adjusted for age, gender, marital status, education level, ever smoking, ever drinking, self-comment about health, hypertension, dyslipidemia, BMI, depressive symptoms, and cognition function at baseline. All variables entered in the models were examined by collinearity diagnostics, and total cholesterol was excluded.

## Data Availability

Data were obtained from the China Health and Retirement Longitudinal Study (CHARLS) research team (http://charls.pku.edu.cn/).

## Supplementary Materials

Supplementary Materials Supplementary Table 1: baseline characteristics among participants completing and not completing cognitive function measurements at 8-year follow-up. Supplementary Table 2: factors associated with cognitive function at follow-up (univariable analyses). Supplementary Table 3: eight-year association between baseline diabetic status (using the WHO diagnostic criteria) and subsequent cognitive function.
