# Supplementary material for: Longitudinal Associations of Newly Diagnosed Prediabetes and Diabetes with Cognitive Function among Chinese Adults Aged 45 Years and Older
Source: J Diabetes Res. 2022 Jul 28;2022:9458646. doi: 10.1155/2022/9458646 (PMC9352492; doi:10.1155/2022/9458646)
Supplement: Supplementary Materials — Supplementary Table 1: baseline characteristics among participants completing and not completing cognitive function measurements at 8-year follow-up. Supplementary Table 2: factors associated with cognitive function at follow-up (univariable analyses). Supplementary Table 3: eight-year association between baseline diabetic status (using the WHO diagnostic criteria) and subsequent cognitive function. [file 9458646.f1.docx]

| **Supplementary Table 1. Baseline characteristics among participants completing and not completing cognitive functionmeasurementsat 8-year follow-up.** | | | |
| --- | --- | --- | --- |
| **Baseline characteristics** | **Completing Cognitive Function at follow-up** | | ***P*-value** |
|  | **Yes (n=6125)** | **No (n=2591)** |  |
| **Gender** |  |  |  |
| Male | 2896 (47.3) | 1091 (42.1) | <0.001 |
| Female | 3225 (52.7) | 1492 (57.6) |  |
| Missing data | 4 (0.1) | 8 (0.3) |  |
| **Age***, year | 57.36 (8.99) | 62.70 (10.47) | <0.001 |
| **Marital status** |  |  |  |
| Married | 5595 (91.3) | 2095 (80.9) | <0.001 |
| Separated or divorced | 61 (1.0) | 34 (1.3) |  |
| Widowed | 438 (7.2) | 431 (16.6) |  |
| Never married | 27 (0.4) | 25 (1.0) |  |
| Missing data | 4 (0.1) | 6 (0.2) |  |
| **Education level** |  |  |  |
| Primary school or below | 3881 (63.4) | 2072 (80.0) | <0.001 |
| Middle school | 1474 (24.1) | 323 (12.5) |  |
| High school or above | 763 (12.5) | 185 (7.1) |  |
| Missing data | 7 (0.1) | 11 (0.4) |  |
| **Ever smoking** |  |  |  |
| Yes | 2398 (39.2) | 954 (36.8) | 0.042 |
| No | 3726 (60.8) | 1636 (63.1) |  |
| Missing data | 1 (0) | 1 (0) |  |
| **Ever drinking** |  |  |  |
| Yes | 3710 (60.6) | 1647 (63.6) | 0.009 |
| No | 2415 (39.4) | 944 (36.4) |  |
| **Self-comment about health** |  |  |  |
| Good | 1237 (20.2) | 427 (16.5) | <0.001 |
| Fair | 3912 (63.9) | 1572 (60.7) |  |
| Poor | 976 (15.9) | 592 (22.8) |  |
| **Hypertension** |  |  |  |
| Yes | 1352 (22.1) | 682 (26.3) | <0.001 |
| No | 4755 (77.6) | 1896 (73.2) |  |
| Missing data | 18 (0.3) | 13 (0.5) |  |
| **Dyslipidemia** |  |  |  |
| Yes | 497 (8.1) | 192 (7.4) | 0.267 |
| No | 5535 (90.4) | 2359 (91.0) |  |
| Missing data | 93 (1.5) | 40 (1.5) |  |
| **Depressive symptoms* (CESD-10)** | 9.01 (5.22) | 9.19 (5.70) | 0.134 |
| **BMI*** (kg/m^2^) | 23.61 (3.59) | 22.99 (3.59) | <0.001 |

Abbreviation: the 10-item Center for Epidemiology Scale for Depression (CESD-10); body mass index, BMI.

^*^: Data was presented in mean (SD).

^c^: The Rao-Scott chi-square test for categorical variables and t-test for continuous variables were used to assess the differences between the groups.

| **Supplementary Table 2. Factors associated with cognitive function at follow-up (univariable analyses)** | | |
| --- | --- | --- |
| **Baseline characteristics** | **Cognitive Function (follow-up, n=6125)** | |
|  | **Unstandardized *β* estimate (95% CI)** | ***P*-value** |
| **Gender** (Male vs. Female) | -1.27 (-1.53~-1.01) | <0.001 |
| **Age** (year)* | -0.19 (-0.21~-0.18) | <0.001 |
| **Marital status** |  |  |
| Married | Ref. |  |
| Separated or divorced | -0.85 (-2.15~0.45) | 0.198 |
| Widowed | -3.22 (-3.72~-2.72) | <0.001 |
| Never married | -4.12 (-6.06~-2.17) | <0.001 |
| **Education level** |  |  |
| Primary school or below | Ref. |  |
| Middle school | 4.32 (4.04~4.60) | <0.001 |
| High school or above | 5.36 (5.00~5.72) | <0.001 |
| **Ever smoking** (Yes vs. No) | -0.46 (-0.73~-0.19) | 0.001 |
| **Ever drinking** (Yes vs. No) | 0.69 (0.42~0.95) | <0.001 |
| **Self-comment about health** |  |  |
| Good | Ref. |  |
| Fair | -0.59 (-0.92~-0.26) | <0.001 |
| Poor | -2.11 (-2.55~-1.68) | <0.001 |
| **Hypertension** (Yes vs. No) | -0.06 (-0.38~0.25) | 0.690 |
| **Dyslipidemia** (Yes vs. No) | -1.16 (-1.64~-0.68) | <0.001 |
| **Depressive symptoms*** | -0.05 (-0.07~-0.02) | <0.001 |
| **Cognition functioning*** | 0.62 (0.59~0.64) | <0.001 |
| **Diabetic Status** |  |  |
| Normal | Ref. |  |
| Prediabetes | -0.08 (-0.41~0.25) | 0.626 |
| Diabetes | -0.60 (-1.05~-0.16) | 0.008 |
| BMI (kg/m^2^)* | 0.19 (0.16~0.23) | <0.001 |
| FBG (mg/dL)* | -0.003 (-0.008~0.002) | 0.299 |
| HbA_1c_ (%)* | -0.208 (-0.423~0.007) | 0.058 |
| BUN (mg/dl)* | -0.04 (-0.07~-0.005) | 0.023 |
| Creatinine (mg/dl)* | 1.77 (1.06~2.48) | <0.001 |
| Total Cholesterol (mg/dl)* | -0.002 (-0.006~0.001) | 0.190 |
| TG (mg/dl)* | 0.002 (0.001~0.004) | <0.001 |
| HDL-c (mg/dl)* | -0.03 (-0.04~-0.02) | <0.001 |
| LDL-c (mg/dl)* | -0.002 (-0.005~0.002) | 0.405 |
| CRP (mg/dl)* | -0.03 (-0.05~-0.01) | 0.001 |
| Hemoglobin (g/dl)* | 0.17 (0.11~0.22) | <0.001 |
| Cystatin C (mg/l)* | -3.41 (-4.04~-2.77) | <0.001 |

Abbreviation: 95% confidence interval, 95% CI; blood urea nitrogen, BUN; triglyceride, TG; HDL cholesterol, HDL-c; LDL cholesterol, LDL-c; High sensitivity C-reactive protein, Hs-CRP.

*: Continuous variable with 1-unit increase.

| **Supplementary Table 3. Eight-year association between baseline diabetic status (using the WHO diagnostic criteria) and subsequent cognitive function** | | | | | | | | | |
| --- | --- | --- | --- | --- | --- | --- | --- | --- | --- |
| **Baseline diabetic status** | **n (%)** | **Cognitive Function (follow-up, n=6125)** | | | | | | | |
|  |  | **Model 1** | | **Model 2** | | **Model 3** | | **Model 4** | |
|  |  | **Unstandardized β estimate (95% CI)** | ***P*-value** | **Unstandardized β estimate (95% CI)** | ***P*-value** | **Unstandardized β estimate (95% CI)** | ***P*-value** | **Unstandardized β estimate (95% CI)** | ***P*-value** |
| **Others^#^** | 4563 (74.5) | Ref. |  |  |  |  |  |  |  |
| **Impaired fasting glucose** | 1011 (16.5) | -0.20 (-0.55~0.16) | 0.281 | -0.70 (-0.38~0.24) | 0.656 | -0.06 (-0.35~0.22) | 0.659 | -0.06 (-0.39~0.28) | 0.749 |
| **Diabetes** | 551 (9.0) | -0.56 (-1.02~-0.10) | 0.018 | -0.53 (-0.93~-0.13) | 0.010 | -0.34 (-0.70~0.03) | 0.073 | -0.57 (-1.05~-0.10) | 0.019 |

Abbreviation: 95% confidence interval, 95% CI; reference, Ref.

Model 1: Unadjusted model.

Model 2: Adjusting for age, gender, marital status, education level, ever smoking, ever drinking, self-comment about health, hypertension, dyslipidemia, BMI, and depressive symptoms at baseline.

Model 3: Adjusting for the variables in Model 1 plus cognitive function at baseline.

Model 4: Adjusting for the variables in Model 2 plus clinical variables including blood urea nitrogen, creatinine, triglycerides, HDL cholesterol, LDL cholesterol, High sensitivity C-reactive protein, hemoglobin, Cystatin C, and HbA_1c_.

#: Others: individuals without prediabetes or diabetes.
